# Descemet Membrane Endothelial Keratoplasty (DMEK) Reduces the Corneal Epithelial Thickness in Fuchs’ Patients

**DOI:** 10.3390/jcm12103573

**Published:** 2023-05-20

**Authors:** Jens Julian Storp, Larissa Lahme, Sami Al-Nawaiseh, Nicole Eter, Maged Alnawaiseh

**Affiliations:** 1 Department of Ophthalmology, University of Muenster Medical Center, 48149 Muenster, Germany; larissa.lahme@ukmuenster.de (L.L.); dr.alnawaiseh@gmail.com (S.A.-N.); nicole.eter@ukmuenster.de (N.E.); 2 Department of Ophthalmology, Klinikum Bielefeld gem. GmbH, 33604 Bielefeld, Germany; maged.alnawaiseh@klinikumbielefeld.de

**Keywords:** optical coherence tomography, Fuchs’ endothelial corneal dystrophy, cornea, epithelial thickness, epithelium, stromal thickness, stroma

## Abstract

Fuchs’ endothelial corneal dystrophy (FECD) is the occurrence of corneal edema due to endothelial cell dystrophy. Descemet membrane endothelial keratoplasty (DMEK) is considered to be the gold standard of treatment. The aim of this study was to investigate the changes in the corneal epithelial thickness of FECD patients before and after DMEK and to compare these results with a healthy control cohort. In this retrospective analysis, 38 eyes of patients with FECD that were treated with DMEK and 35 healthy control eyes received anterior segment optical coherence tomography (OCT; Optovue, XR-Avanti, Fremont, CA, USA). The corneal epithelial thicknesses in different locations were analyzed and compared between the preoperative, postoperative, and control cohorts. The median follow-up time was 9 months. There was a significant degression of the mean epithelial thickness after DMEK in the central, paracentral, and mid-peripheral zones (*p* < 0.01) of the cornea. The total corneal thickness and stromal thickness decreased significantly as well. No significant differences were observed between the postoperative and control cohorts. In conclusion, the FECD patients had an increased epithelial thickness compared to the healthy controls, which decreased significantly after DMEK and reached thickness levels comparable to those of healthy control eyes. This study emphasized the importance of distinguishing between the corneal layers in anterior segment pathologies and surgical procedures. Moreover, it accentuated the fact that the structural alterations in FECD extend beyond the corneal stroma.

## 1. Introduction

Fuchs’ endothelial corneal dystrophy (FECD) describes the progressive loss of corneal endothelial cell function, which leads to the swelling of the cornea and causes visual impairment and long-term structural changes to the different corneal layers [1]. Endothelial cell loss typically first manifests in the fourth and fifth decades of life and, if left untreated, often results in blindness [1]. FECD occurs more often in females than males [2]. With a prevalence of 4% in the population aged 40 years and older in the United States of America, it is less common in Middle Eastern and Asian countries [2,3]. At the same time, it is estimated that FECD will affect more than 400 million people globally by 2050 [4]. Currently, Descemet membrane endothelial keratoplasty (DMEK) is considered to be the gold standard for the treatment of patients suffering from FECD. In this form of lamellar keratoplasty, the Descemet membrane and corneal endothelium of an eye with FECD are surgically removed and replaced with a healthy donor transplant. This isolated replacement of unhealthy corneal tissue with a healthy transplant has been shown to be more effective than previous surgical techniques, as DMEK is associated with a faster visual recovery and improved refractive and visual outcomes, as well as fewer graft rejections and postoperative supplemental medication [2,5,6]. A number of studies have demonstrated the lasting long-term rehabilitation of patients having received DMEK [7,8,9,10].

**Figure 1:**
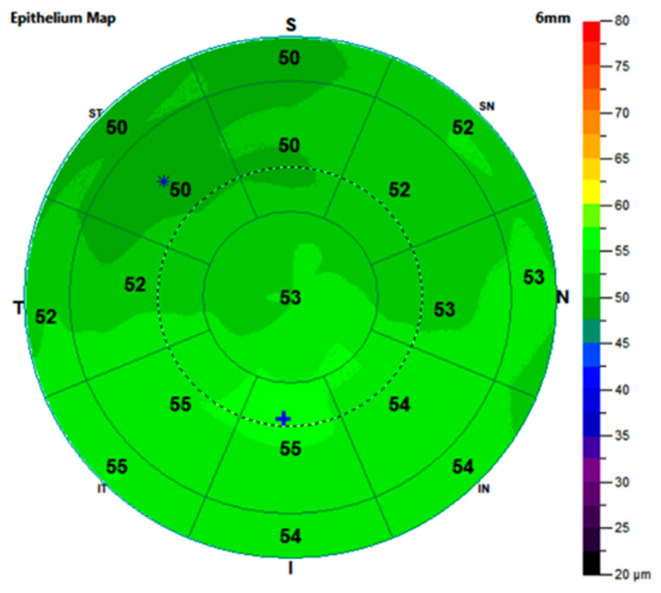
An exemplary representation of the epithelial thickness map of a healthy control eye with its sectors and respective values calculated automatically by the instrument software. S: superior, I: inferior, T: temporal, N: nasal, ST: superior temporal, SN: superior nasal, IT: inferior temporal, and IN: inferior nasal, *: location in which the epithelium is thinnest, +: location in which the epithelium is thickest.

In everyday clinical routine, the evaluation of patients with FECD usually involves a slit lamp examination and Scheimpflug photography. Optical coherence tomography (OCT) has become an integral part of everyday ophthalmologic practice, especially in the field of retinal diagnostics. Nowadays, the use of OCT is also becoming increasingly important for corneal diagnostics. Anterior segment OCT is a non-contact, non-invasive diagnostic tool that uses high-wavelength light to render cross-sectional maps of the cornea and other structures of the anterior segment of the eye. The latest advances have allowed for histology-like investigations of the different corneal layers, providing new insights into the etiology and development of corneal diseases, as well as into the effectiveness of corneal surgeries [11]. Due to its very high resolution, OCT examination allows for an accurate visualization of the different corneal layers. Automatic segmentation algorithms also make it possible to measure the individual corneal layers and display them as color-coded thickness maps in their different localizations (Figure 1) [12]. OCT allows for an analysis of the changes in the corneal structure of FECD eyes and eyes treated with DMEK [13,14,15].

Several studies have investigated the impact of DMEK on corneal tissue on a structural scale. The postoperative effects of DMEK are most apparent in the reduction in the central corneal thickness, which results from the de-swelling function of the transplanted endothelial cells [16,17,18]. While changes in the stromal thickness or total corneal thickness have been well described in DMEK research, changes in the corneal epithelial layer have not yet been investigated. As they are of great relevance for corneal topography, the adequate thickness and function of epithelial cells are crucial for corneal morphology and thus for the visual system as a whole [19]. A functioning epithelium is also crucial for underlying tissues such as the corneal stroma, as this has been shown to protect against ultraviolet radiation damage [20].

The aim of this work was to investigate the changes in corneal epithelial thickness after DMEK in FECD patients.

## 2. Materials and Methods

This study adhered to the tenets of the Declaration of Helsinki and complied with the regulations issued by the local ethics committee of the Medical Association of Westfalen-Lippe and the Westphalian Wilhelms University Münster. Informed consent was waived due to the retrospective nature of the study. To address the potential ethical concerns, the data were exclusively retrieved from patients aged 18 years or older. In addition, only data relevant for the analyses of this study were anonymized and reviewed. Additional information was not collected.

The data in this retrospective study were retrieved from the electronic medical records of patients with FECD who had received anterior segment OCT imaging before and after DMEK, as well as of healthy control patients who had also received anterior segment OCT imaging at the Department of Ophthalmology at the University Hospital Münster, Germany. General patient information, as well as data from refractive eye exams, findings from routine slit-lamp examinations, dates of surgery, intraoperative or postoperative complications, current and previous ocular medications, ophthalmological histories, and histories of previous ocular surgeries were collected. The anterior segment OCT measurement results were extracted using an automated export function of the device and then reviewed manually. In order to reduce potential bias, only patients with completely filled out electronic patient files were drawn into the study. An electronic patient file was considered complete if it included details on all of the aforementioned parameters. All the surgeries were performed by the same surgeon. The postoperative evaluations of the patients who had undergone DMEK were performed at least 3 months after surgery.

The examination methods were identical for the control and FECD cohorts. To be included, the FECD patients had to have a definitive diagnosis of FECD in the studied eye. Patients who suffered from other corneal pathologies or had undergone previous surgery in the studied eye were excluded. If patients required a re-bubbling of the transplant, the OCT measurements were obtained from scans that were generated at least 3 months after the re-bubbling. The control cohort patients were not allowed to exhibit any form of corneal pathology. This was confirmed by slit-lamp examinations and evaluations of the cornea using anterior segment OCT. In addition, control patients were only eligible for inclusion if they had no history of corneal disease, corneal surgery, or corneal laser therapy. Control patients were excluded if they took eye drops known to affect corneal thickness, such as topical steroids. If the visual acuity was low for these patients, this had to be attributable to diseases unrelated to the cornea, for example, to retinal pathologies. All the control and FECD patients were of Caucasian descent and no participant wore contact lenses on a regular basis.

### 2.1. Surgical Procedure

On the day prior to the DMEK surgery, an iridotomy is performed at the 6 o’clock position of the patient’s iris with a neodymium-doped yttrium aluminum garnet (Nd:YAG) laser to avoid postoperative angle blockage with intraocular pressure decompensation. The DMEK lamella is obtained preoperatively from a donor cornea by the same surgeon. The operation is performed under general anesthesia. The surgical steps during DMEK conducted at our clinic are as follows.

Two side ports are made at the 2 and 10 o’clock positions. The preexisting iridotomy is spread open gently and the anterior chamber is then inflated with air, followed by a “descemetorhexis”. An inverted Sinskey hook (Geuder AG, Heidelberg, Germany) is used to score the Descemet membrane and remove it from the posterior stroma. To insert the graft, a 2.8 mm incision is created at the limbus. Before being injected into the anterior chamber, the donor Descemet roll is stained with a 0.1% trypan blue solution (Vision Blue, D.O.R.C. International) and then gently sucked into a glass injector (DMEK-Inserter, Geuder). By using indirect manipulation with air and a balanced salt solution, the graft is turned in such a way that the endothelial side faces downwards and is then unrolled by gently tapping on the cornea and varying the depth of the anterior chamber. It is further stretched out gently to even out any irregularities. An air bubble is then injected beneath the graft to push it onto the posterior corneal stroma of the recipient. After the anterior chamber has been completely filled with air for ten to twenty minutes, an air–liquid exchange is performed to pressurize the eye, while maintaining an air bubble the size of approximately 80% of the anterior chamber. The patient is then required to stay in a supine position for at least four hours.

### 2.2. Corneal Imaging

The OCT images for all the groups were performed under the same conditions, with an expert examiner using the Optovue XR-Avanti system (Optovue, XR Avanti, Fremont, CA, USA). The OCT measurements were conducted in the same room, in the same lighting conditions. The epithelial thickness was evaluated using an anterior segment adapter from the same manufacturer and was automatically differentiated by the instrument software (AngioAnalytics, version 2017.1.0.151, Optovue, Fremont, CA, USA). The corneal pachymetry analysis of the instrument software provided a color-coded corneal thickness map with divisions in different locations of the corneal center. The corneal epithelial thickness was reported in the central 2 mm, paracentral 2–5 mm, and mid-peripheral 5–6 mm around the apex. The resulting ring-like geometric areas for the paracentral region and mid-periphery were further divided into 8 sectors and the values for these were reported separately. Altogether, each measurement consisted of and displayed 17 individual sectors (Figure 1).

Due to a lack of universally acknowledged reference values for the corneal epithelial thickness measured using anterior segment OCT, the values of the control cohort were compared to the postoperative outcomes to investigate whether DMEK could achieve epithelial thickness normalization.

The average thickness values for the paracentral and mid-peripheral rings were calculated. The thickness values for the center, average paracentral region, and average periphery were compared among the preoperative, postoperative, and control groups. Equivalently, a sectoral approach investigated the individual subregions of the paracentral and mid-peripheral rings.

### 2.3. Statistical Analysis

The data were entered into a Microsoft Excel 2010 spreadsheet and statistically analyzed using SPSS^®^ Statistics 28 for Windows (IBM Corporation, Somers, NY, USA). The data were tested for normal distribution using the Shapiro–Wilk test. As this normal distribution could not be confirmed, the two groups were compared using the Mann–Whitney U test and the degree of correlation between the two variables was expressed as Spearman’s correlation coefficient. The data are reported as median and interquartile ranges (25% quartile; 75% quartile).

A post hoc analysis was performed using G*Power 3.1 (Heinrich-Heine-University Düsseldorf, Germany, www.gpower.hhu.de). For an α of 0.05, we determined a power of 0.99 for the pre- and post-DMEK comparison. The inferential statistics are intended to be exploratory (hypotheses generating), not confirmatory. The comparison-wise type-I error rate was controlled instead of the experiment-wise error rate. Due to the exploratory nature of the study, no adjustment was made for multiple testing. Exploratory *p*-values ≤ 0.05 were considered to be statistically noticeable.

## 3. Results

In total, 38 eyes of 38 FECD patients were followed-up postoperatively and 35 eyes of 35 healthy controls were included in the comparative analyses. The median follow-up time was 8.9 (5.3; 13.9) months. The characteristics of the study population are summarized in Table 1.

There were no significant differences in the ages or spherical equivalents between the patients with FECD and the healthy controls (age: *p* = 0.22, spherical equivalent: *p* = 0.73). Neither the ratio between the right to left eyes (*p* = 0.43), nor the ratio between the male to female participants (*p* = 0.72) were noticeably different between the FECD and control cohorts. Visual acuity increased significantly after surgery (*p* < 0.01) (Table 1).

Of the 38 eyes with FECD, 7 were treated with phakic DMEK, 9 with Triple-DMEK, and 22 with pseudophakic DMEK. Five eyes required re-bubbling. Of these, 2 patients received re-bubbling during the first week after surgery and 3 received re-bubbling within 3 weeks after the initial DMEK surgery.

**Figure 2:**
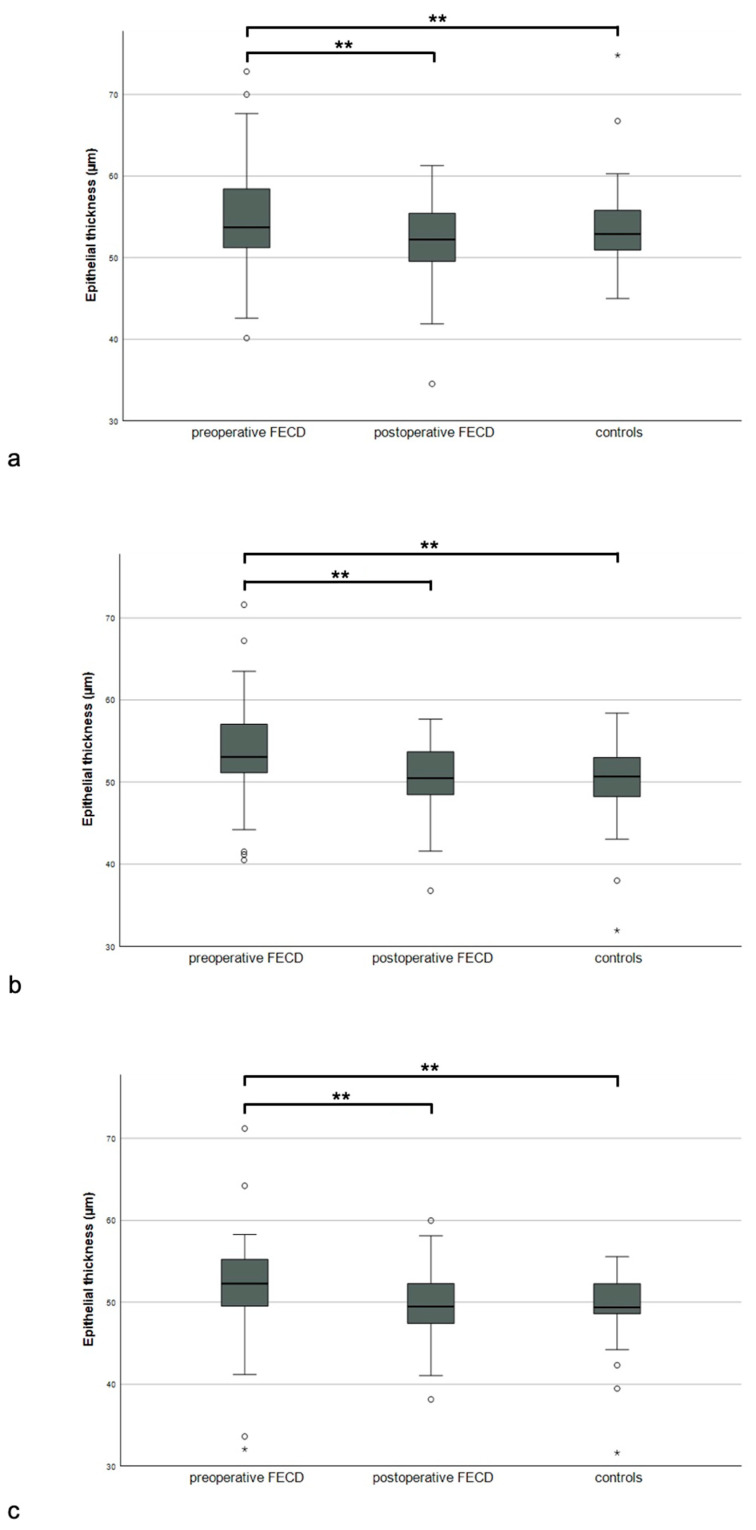
Epithelial thickness of the preoperative, postoperative, and control groups in central ((**a**), 2 mm), paracentral ((**b**), 2–5 mm), and mid-peripheral ((**c**), 5–6 mm) corneal zones, * for *p* < 0.05, ** for *p* < 0.01.

The central, average paracentral, and average mid-peripheral corneal epithelial thickness values were noticeably greater in the preoperative FECD eyes than the healthy control eyes. Similarly, the eyes of the preoperative FECD group showed higher values than the postoperative eyes for the central, average paracentral, and average mid-peripheral corneal epithelial thicknesses, meaning that the corneal epithelial thickness decreased noticeably after DMEK surgery in these sectors. There were no differences between the epithelial thickness values for the postoperative and control eyes (Figure 2, Table 2).

Table 2 summarizes the results for the corneal thickness in the pre- and postoperative eyes, as well as in the control eyes, for the central, average paracentral, and average mid-peripheral total, stromal, and epithelial corneal thickness values. While the total and stromal thicknesses were the highest preoperatively, both decreased significantly after DMEK in all the sectors. There were no differences between the postoperative group and the controls in either the total, stromal, and epithelial sectors.

In the sectoral analysis, the median epithelial thickness was noticeably greater in the preoperative rather than the postoperative patients for all the sectors, except for the mid-peripheral inferior-temporal sector. The sectoral comparison of the postoperative eyes to the control eyes did not reveal any noticeable differences (Table 3).

**Figure 3:**
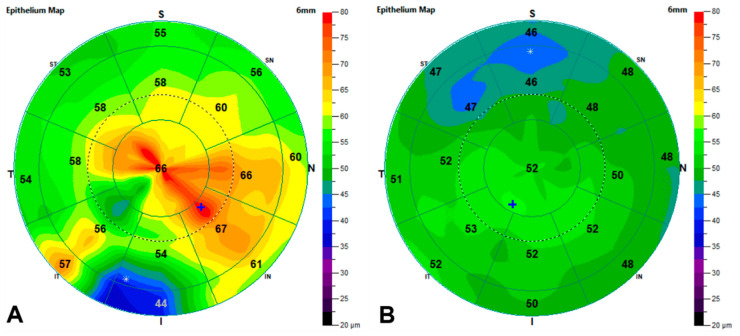
Epithelial thickness maps before and after DMEK on the right eye of an FECD patient. Measurements were taken 303 days apart. (**A**): preoperative epithelial thickness map. (**B**): postoperative epithelial thickness map. S: superior, I: inferior, T: temporal, N: nasal, ST: superior temporal, SN: superior nasal, IT: inferior temporal, and IN: inferior nasal, *: location in which the epithelium is thinnest, +: location in which the epithelium is thickest.

Figure 3 exemplifies the changes in the epithelial thickness after DMEK in one eye of the FECD cohort.

## 4. Discussion

Prior to surgery, the patients with FECD showed an increased epithelial thickness compared to the healthy controls. This epithelial thickness decreased significantly after the DMEK surgery in this study and approached the values of healthy control eyes.

In vivo layer-specific measurements of the human cornea have been conducted using various imaging modalities. Former studies have applied technologies, such as confocal laser scanning microscopy [21] or very high frequency (VHF) digital ultrasound scanning [22], to investigate the corneal epithelium. Lately, anterior segment OCT has emerged as a promising new technology. High-resolution images of the cornea obtained with OCT technology provide a detailed visualization of the corneal epithelium [23]. In recent years, studies have shown the good reproducibility of epithelial thickness measurements obtained with OCT on healthy subjects and patients with various corneal diseases, as well as on patients receiving corneal treatments [24,25].

This study is the first to investigate changes in the epithelial thickness in FECD patients treated with DMEK using anterior segment OCT. We analyzed the measurements of 38 FECD patients who received DMEK, analyzed the epithelial thickness before and after surgery, and compared the results with those of a control cohort.

In this study population, the epithelial thickness was greatest in patients with FECD before DMEK treatment. After surgery, the epithelial thickness significantly decreased and approached thickness levels comparable to the values of healthy individuals. This might be indicative of a trend towards normalization in the corneal epithelial thickness after DMEK surgery in Fuchs’ patients. The epithelial thickness of the control cohort was comparable to the reference values that have been reported in the literature [22].

Although some studies have identified increases in the thicknesses of specific corneal layers in patients with FECD using OCT, such as the endothelium–Descemet membrane complex, there is currently a lack of data on the influence of FECD on the corneal epithelium [26]. Eleiwa et al. investigated alterations in the total corneal thickness, the endothelium–Descemet complex of the central cornea, and the central to peripheral total corneal thickness ratio in patients with FECD compared to controls using OCT. They described a significant increase in all of these parameters in the patients with FECD [26].

Currently, there is also a lack of information about the effect of lamellar keratoplasties on the corneal epithelium. Studies have traditionally focused on the changes in the total corneal thickness in patients with lamellar keratoplasty, rather than on the individual corneal layers. Regarding the changes in the corneal thickness after DMEK, most studies have compared the central corneal thickness pre- and postoperatively and did not differentiate between the individual corneal layers. This is, in part, attributed to the fact that high-resolution anterior segment imaging technology, which is required for the precise segmentation of the individual corneal layers, is not generally available, and that the software capabilities of early OCT devices might have limited layer-specific analyses. For example, Agha et al. showed a significant decrease in the central corneal total thickness using a rotating Scheimpflug camera system [27]. Other studies have investigated the effect of DMEK on various parameters in patients with FECD using anterior segment OCT. Most of these tomography-based studies have described a significant decrease in the central corneal thickness in patients with FECD after lamellar keratoplasty [17,27,28]. Machalinska et al. applied swept-source anterior segment OCT to analyze the central corneal thickness before and after DMEK. They reported a significant reduction in the central corneal thickness after DMEK [28]. Similarly, Brockmann et al. also observed a significant reduction in the central corneal thickness after DMEK in an anterior segment OCT study [17]. The authors reported a postoperative corneal thickness of 529 µm, which is very similar to the postoperative thickness values reported in this study (527 µm), as well as to the values reported by Agha et al. (523 µm) [27]. Machalinska et al. even distinguished certain time points in their follow-up period, describing that the reduction in the central corneal thickness after DMEK was noticeable from the first month after surgery and continued for up to 12 months after the transplantation [28]. The data from this trial suggest that this reduction in the total corneal thickness should be attributed, in part, to a de-swelling of the corneal epithelium after DMEK.

Despite the number of trials investigating the changes in the cornea using high-resolution imaging, the effects of DMEK on the individual corneal layers, such as the corneal epithelium, remain unclear. The importance of the differentiation between the corneal layers should not be underestimated, as changes in the different corneal layers can cause distinct symptoms. For instance, Okumura et al. reported that an increase in the epithelial thickness of FECD patients was correlated with a decrease in contrast sensitivity. They highlighted the relevance of a normal epithelial thickness for patients’ vision-depending activities, such as driving and reading [19]. In addition, the generation of data on the changes in the individual corneal layers associated with lamellar keratoplasties is of great interest, since the optimization of refractive outcomes has become increasingly relevant in clinical practice [29]. Understanding the changes in the individual corneal layers related to lamellar surgery might further increase the knowledge of postoperative deviations in refraction, as have been reported by a number of trials [30,31,32]. A normal corneal epithelium and corneal stroma contribute not only to the correct refraction, but also to the physiological functioning of the cornea as an organ [33], which highlights the importance of layer-specific analyses of this tissue.

### Limitations

This study reports findings in the 6 mm sector around the corneal apex. We are therefore unable to comment on changes beyond this 6 mm boundary. Changes related to DMEK treatment in the corneal periphery might be of importance for postoperative refractive outcomes [34]. Previous studies have discussed that a remaining peripheral swelling might cause a postoperative hyperopic shift in patients, as it steepens the posterior curvature of the cornea [16,35,36]. To what extent these changes are attributable to changes in the individual corneal layers remains unknown. Further OCT studies encompassing measurements of the corneal periphery are needed to investigate the changes in these regions. These studies should preferably be conducted on either the right or left eyes only, in order to rule out possible bias related to the surgical procedure.

Due to the retrospective nature of this study, we are unable to comment on prospective estimates of the thickness changes over longer or shorter periods than the one covered in this trial.

Though the comparability of the OCT measurements to some other imaging modalities, such as VHF digital ultrasounds, has been demonstrated [37], comparisons to other studies using different technologies should be done with caution.

Furthermore, we did not differentiate the patients based on the severity of their FECD. The de-swelling effect of DMEK on the corneal epithelium might be less apparent in patients with early-stage FECD than patients with advanced FECD. Further studies investigating the effect of lamellar keratoplasties on the epithelial thickness in different disease stages are needed.

## 5. Conclusions

To summarize, this study shows that the epithelial thickness in patients with FECD is increased in the paracentral and mid-peripheral zones in comparison to healthy control eyes. After DMEK, this epithelial thickness decreases significantly in the central, paracentral, and mid-peripheral sectors and reaches values comparable to those of healthy control eyes. This trial underscores the significance of differentiating the corneal layers in anterior segment pathologies, as well as surgery, and highlights the presence of reversible structural changes in FECD that go beyond the corneal stromal layer.

## Figures and Tables

**Table 1:** Characteristics of the study population. Data are reported as median (25% quartile; 75% quartile).

	FECD Cohort		Control Cohort
	Preoperative	Postoperative	
n (patients)	38		35
n (eyes)	38		35
age (years)	67.24 (59.79; 74.70)	67.92 (60.14; 75.22)	70.31 (62.93; 77.02)
gender (m:f)	19:19		16:19
eye (r:l)	16:22		18:17
visual acuity [logMAR]	0.50 (0.70; 0.40)	0.20 (0.30; 0.10)	0.10 (0.20; 0.00)
spherical equivalent [D]	0.00 (−1.38; 0.94)	−0.35 (−1.12; 0.34)	−0.25 (−1.18; 0.45)

n: number, m: male, f: female, r: right, l: left, logMAR: Logarithm of the Minimum Angle of Resolution, and D: diopters.

**Table 2:** Statistical results for the central 2 mm, paracentral 2–5 mm, and mid-peripheral 5–6 mm corneal thickness values for pre- and postoperative eyes of FECD patients and control eyes (*p* values < 0.05 are highlighted in bold).

Localization		Corneal Thickness Median (25% Quartile; 75% Quartile)			*p* Values		
		Pre-Operative	Post-Operative	Controls	Pre- vs. Post-Operative	Pre-Operative vs. Controls	Post-Operative vs. Controls
Total	Central	617.70 (564.21; 672.34)	527.68 (484.75; 555.53)	532.31 (501.71; 571.28)	**<0.01**	**<0.01**	0.28
	Paracentral	608.28 (576.53; 677.58)	551.22 (506.39; 576.94)	549.53 (519.94; 590.16)	**<0.01**	**<0.01**	0.51
	Mid-peripheral	616.48 (592.23; 676.66)	579.65 (535.20; 607.68)	577.36 (543.73; 614.87)	**<0.01**	**<0.01**	0.71
Stroma	Central	563.46 (510.17; 617.01)	478.83 (430.75; 499.64)	466.86 (439.32; 507.78)	**<0.01**	**<0.01**	0.75
	Paracentral	554.71 (520.82; 622.22)	502.91 (455.57; 526.95)	481.18 (460.00; 526.18)	**<0.01**	**<0.01**	0.96
	Mid-peripheral	562.38 (495.85; 620.82)	532.41 (483.37; 557.18)	505.23 (483.10; 552.22)	**<0.01**	**<0.01**	0.68
Epithelium	Central	54.42 (51.79; 60.20)	52.10 (48.68; 55.41)	52.60 (50.67; 54.59)	**<0.01**	**0.01**	0.51
	Paracentral	53.67 (51.12; 57.85)	50.25 (47.34; 53.62)	50.26 (48.20; 52.67)	**<0.01**	**<0.01**	0.87
	Mid-peripheral	52.55 (49.55; 55.87)	48.95 (46.68; 52.23)	49.25 (48.58; 51.97)	**<0.01**	**0.02**	0.54

**Table 3:** Statistical results of the comparison between paracentral and mid-peripheral epithelial thicknesses, according to subregions between pre- and postoperative FECD and control eyes (*p* values < 0.05 are highlighted in bold).

Localization		Epithelial Thickness Median (25% Quartile; 75% Quartile)			*p*-Value	
		Preoperative	Postoperative	Controls	Pre- vs. Postoperative	Post-Operative vs. Controls
paracentral	temporal	53.07 (49.46; 57.32)	50.17 (47.05; 53.50)	49.67 (47.55; 52.43)	**0.01**	0.96
	superior-temporal	52.00 (48.61; 56.19)	49.02 (45.52; 51.37)	48.90 (47.22; 50.79)	**<0.01**	0.90
	superior	51.59 (49.55; 55.70)	48.00 (45.94; 51.63)	48.12 (46.83; 50.90)	**<0.01**	0.90
	superior-nasal	52.70 (49.70; 56.74)	49.96 (46.78; 52.84)	49.16 (47.50; 51.84)	**0.01**	0.67
	nasal	53.49 (50.50; 57.57)	49.84 (47.92; 53.95)	50.57 (48.18; 53.92)	**0.02**	0.83
	inferior-nasal	55.13 (52.46; 58.63)	52.00 (48.17; 54.91)	51.48 (49.87; 54.04)	**<0.01**	0.76
	inferior	55.07 (53.09; 58.84)	53.15 (49.20; 55.91)	51.50 (49.80; 55.09)	**<0.01**	0.68
	inferior-temporal	54.24 (51.16; 58.31)	52.65 (48.07; 55.79)	51.32 (48.44; 54.24)	**0.04**	0.39
mid-peripheral	temporal	50.96 (49.43; 54.04)	49.41 (46.11; 52.18)	49.70 (47.75; 51.86)	**0.01**	0.72
	superior-temporal	49.92 (46.97; 53.81)	47.35 (43.54; 50.05)	48.12 (44.94; 49.74)	**<0.01**	0.87
	superior	50.38 (47.55; 53.19)	46.34 (43.00; 49.33)	46.22 (43.90; 49.34)	**<0.01**	0.83
	superior-nasal	52.19 (49.34; 55.64)	48.89 (44.79; 51.68)	46.79 (45.31; 52.64)	**<0.01**	0.62
	nasal	54.02 (49.79; 56.22)	50.43 (46.58; 53.38)	50.37 (48.14; 52.63)	**0.05**	0.67
	inferior-nasal	54.19 (50.64; 56.98)	51.39 (47.80; 54.70)	51.57 (49.96; 53.48)	**0.01**	0.69
	inferior	54.68 (49.55; 57.55)	52.03 (48.16; 54.32)	51.57 (49.16; 53.40)	**0.02**	0.98
	inferior-temporal	52.55 (49.81; 56.26)	51.78 (47.95; 54.37)	52.76 (50.77; 53.83)	0.14	0.22

## Data Availability

Not applicable.
